# Pramipexole treatment and substance use disorder risk in unipolar and bipolar depression: a Swedish nationwide register study

**DOI:** 10.3389/fpsyt.2026.1919633

**Published:** 2026-08-10

**Authors:** Sara Lindström, Mirjam Wolfschlag, Anders Håkansson, Jonas Berge

**Affiliations:** 1 Department of Clinical Sciences, Lund, Psychiatry, Lund University, Lund, Sweden; 2 Department of Psychiatry, Lund, Skåne University Hospital, Lund, Sweden; 3 Department of Psychiatry, Malmö, Skåne University Hospital, Malmö, Sweden

**Keywords:** bipolar disorder, major depressive disorder, pharmacoepidemiology, pramipexole, register study, substance use disorder

## Abstract

**Background:**

Over the past decades, evidence supporting the antidepressant efficacy of the dopamine agonist pramipexole as an augmentation strategy in mood disorders has accumulated. While dopaminergic treatments have been associated with behavioural adverse effects, the relationship between pramipexole treatment and incident substance use disorder in psychiatric populations remains poorly understood.

**Aims:**

To examine the association between cumulative pramipexole exposure and incident substance use disorder among individuals with unipolar depression or bipolar disorder.

**Methods:**

We utilised data from Swedish national registries to conduct a nationwide cohort study including individuals with unipolar depression or bipolar disorder treated in specialised mental health care (n = 3105) who were prescribed pramipexole. Cumulative pramipexole exposure was modelled longitudinally and analysed as a time-varying variable. The primary outcome was incident substance use disorder identified in specialised healthcare registers. Associations were examined using Cox proportional hazards models, with analyses separated by diagnostic subgroup.

**Results:**

High cumulative exposure to pramipexole was associated with an increased risk of incident substance use disorder in the overall cohort (aHR 1.51, 95% CI 1.02–2.23), whereas intermediate exposure was not associated with elevated risk. In stratified analyses, the association was particularly pronounced among individuals with bipolar disorder, where high cumulative exposure was associated with more than a twofold increased risk (aHR 2.54, 95% CI 1.25–5.16). No significant association was observed among individuals with unipolar depression.

**Conclusions:**

High cumulative exposure to pramipexole was associated with increased risk of incident substance use disorder in individuals with mood disorders, with the strongest associations observed in bipolar disorder. Although no causal inference can be made due to the observational design, these findings raise the possibility that prolonged pramipexole exposure may be associated with increased risk of substance use disorder in some psychiatric populations, particularly among individuals with bipolar disorder.

## Introduction

### Mood disorders, substance use, and reward dysregulation

Mood disorders, including unipolar depression and bipolar disorder, are associated with substantial morbidity and mortality, with suicide representing a leading cause of premature death in these populations (1). Further, mood disorders are strongly associated with an increased risk of substance use disorders (SUD), contributing substantially to morbidity, mortality, and impaired treatment outcomes (2). In particular, bipolar disorder has consistently been linked to elevated rates of alcohol and drug use disorders, earlier onset of substance use, and more severe clinical courses, suggesting a heightened vulnerability to reward-driven and disinhibited behaviours (3).

### Pramipexole in mood disorders and behavioural adverse effects

Dopaminergic neurotransmission plays a central role in reward processing, motivation, and reinforcement learning. Dysregulation of mesolimbic dopamine pathways has been implicated both in mood disorders and in substance use disorders (4). Consequently, pharmacological interventions targeting dopaminergic systems have attracted increasing interest as potential treatments for treatment-resistant depression.

Pramipexole is a dopamine agonist with high affinity for D2 and D3 receptors, both of which are expressed in limbic regions involved in reward processing (5). While originally developed for Parkinson’s disease and restless legs syndrome, an accumulating body of evidence supports its antidepressant efficacy as an augmentation strategy in treatment-resistant unipolar and bipolar depression. Early randomised controlled trials demonstrated beneficial effects in bipolar depression (6, 7) and treatment-resistant major depressive disorder (8). Subsequent observational studies and systematic reviews have further supported its effectiveness and tolerability in clinical practice (9, 10). More recently, larger randomised controlled trials have demonstrated clinically meaningful improvements in depressive symptoms and anhedonia, further strengthening the evidence base for pramipexole in mood disorders (11–13). Consequently, pramipexole has gained increasing clinical interest as an off-label treatment option for patients who do not respond adequately to conventional therapies.

Dopamine agonists have been associated with behavioural adverse effects, particularly impulse control disorders such as pathological gambling, compulsive shopping, compulsive sexual behaviour, and binge eating (14–16). These adverse effects are well documented in Parkinson’s disease populations, although evidence from psychiatric populations remains more limited. Importantly, substance use disorders and impulse control disorders share several neurobiological mechanisms, including altered reward processing, impaired inhibitory control, and maladaptive reinforcement learning (4, 17). From a theoretical perspective, sustained dopaminergic stimulation may therefore influence vulnerability not only to behavioural addictions but also to substance-related addictive disorders.

However, despite growing interest in pramipexole as a treatment for mood disorders, the potential association between pramipexole exposure and incident substance use disorder remains poorly understood. Recent studies have increasingly incorporated assessments of impulse-control behaviours and related behavioural adverse effects, including gambling, compulsive behaviours, and treatment-emergent mood elevation (12, 13). While some studies have reported increased impulsive behaviours during treatment, incident substance use disorder has not, to our knowledge, been examined as a primary or secondary outcome in clinical studies of pramipexole treatment for mood disorders. Furthermore, both McAllister-Williams et al. and Ventorp et al. excluded individuals with current substance use disorders/substance misuse at study entry, limiting the possibility of evaluating addiction-related outcomes in populations at elevated baseline risk. Substance use disorder has not emerged as a reported adverse outcome in published studies of pramipexole in psychiatric populations. However, existing studies have primarily focused on antidepressant efficacy, short-term safety, and behavioural adverse effects during relatively brief periods of follow-up. Nationwide healthcare registers offer a complementary pharmacoepidemiological approach by enabling the study of uncommon or delayed outcomes in large populations treated under real-world conditions and over extended follow-up periods.

The present nationwide cohort study aimed to investigate the association between cumulative pramipexole exposure and the risk of incident substance use disorder among individuals with mood disorders. We further examined whether this association differed between unipolar depression and bipolar disorder and whether the findings indicated an exposure–response pattern.

## Methods

In this longitudinal population-based register study, we utilised Swedish national health registers to investigate substance use disorder risk associated with pramipexole treatment among patients with unipolar and bipolar depression. We extracted data from three comprehensive national databases maintained by the Swedish National Board of Health and Welfare for the period 2006-2021: the Swedish Prescribed Drug Register (PDR), the National Patient Register (NPR), and the Cause of Death Register (CDR).

The PDR provides complete coverage of all prescription medications dispensed at Swedish pharmacies since July 1, 2005, including detailed information on dispensing dates, drug strength, package sizes, quantities dispensed, and prescription renewals (18). The NPR is based on visits in specialised health care and comprises two components: The inpatient register, which has documented psychiatric diagnoses since 1973 with nationwide coverage achieved in 1987, and the outpatient register, established in 2001 with approximately 90% coverage by 2010 and 99% coverage by 2017 (19, 20). The CDR maintains comprehensive mortality records, including dates and underlying causes of death (21) Data for diagnostic inclusion and exclusion criteria were obtained from both inpatient and outpatient components of the NPR as well as from the PDR.

The source population included all individuals who received at least one pramipexole prescription (ATC code N04BC05) between July 1, 2005, and December 31, 2021. In order to ascertain a minimum of baseline data as well as exclude patients with ongoing pramipexole treatment, we excluded patients who had their first prescription prior to July 1, 2006. Inclusion criteria required: (1) documented mood disorder diagnosis (unipolar depression: F32-F33; bipolar disorder: F30-F31) recorded in the NPR at the index date or within 365 days prior to that date, and (2) prescription for antidepressant medication (ATC codes N06A) or lithium (ATC code N05AN01) at the index date or within 365 days prior to that date.

Exclusion criteria eliminated patients with: (1) baseline substance use disorders (ICD-10 codes F10-F19), (2) baseline alcohol use disorder medication treatment (ATC codes N07BB) or opioid use disorder medication treatment (ATC codes N07BC, not including buprenorphine when used for pain management, under the ATC code N02AE01), and (3) documented Extrapyramidal and movement disorders including Parkinson’s disease (ICD-10 codes G20-G26). Extrapyramidal and movement disorders were excluded to focus on a sample of patients without dopaminergic neurodegeneration, treated for mood disorders in specialist psychiatry, i.e. the target group for pramipexole treatment as augmentation to standard treatment for depressive disorders, or for other somatic indications like fibromyalgia or restless legs syndrome without a specialist diagnosis. The index date was defined as the first pramipexole prescription date, with baseline characteristics assessed using all available historical data in the registers prior to this date.

We quantified pramipexole exposure as cumulative dose calculated from prescription records including dispensed quantities, tablet strengths, and package sizes. Cumulative exposure represented the total amount of pramipexole prescribed for each patient at the time of each outcome event in the cohort. A standardised daily dose of 1.0 mg was established as the reference unit for dose calculations, based on the typical therapeutic range of 0.5-1.5 mg daily when pramipexole is used as augmentation for depressive disorders in randomized controlled trials (10). Cumulative exposure was computed as a time-varying covariate, updated at each outcome event time throughout the cohort follow-up period.

Due to the highly skewed distribution of cumulative doses, we categorised exposure into clinically meaningful dose categories based on average daily dose equivalents per year: low exposure (0 to <1 standardised daily dose equivalent per year), intermediate exposure (1 to <2 dose equivalents per year), and high exposure (2+ dose equivalents per year).

The primary outcome was incident substance use disorder, defined as either: (1) a new diagnosis of substance use disorder recorded in specialised healthcare (ICD-10 codes F10–F19), or (2) initiation of pharmacological treatment for alcohol or opioid use disorder (ATC codes N07BB and N07BC, respectively). Buprenorphine prescribed for pain management under ATC code N02AE01 was not included. Treatment indicators were included because the National Patient Register does not capture diagnoses made exclusively in primary care, whereas the Prescribed Drug Register includes medications dispensed across healthcare settings. Follow-up continued from the index date until the first substance use disorder outcome, death, or 31 December 2021, whichever occurred first. Demographic variables included gender, age at index date, and mortality status. We also included comorbid anxiety disorders as a covariate, given their established association with substance use disorder risk and high prevalence in affective disorder populations. Comorbid anxiety disorders were assessed from diagnoses registered in the NPR and defined as ICD-10 codes F40-F49 (“Anxiety, dissociative, stress-related, somatoform and other nonpsychotic mental disorders” according to ICD-10), which we refer to simply as anxiety disorders throughout this study.

### Statistical methods

Descriptive analyses compared baseline characteristics between patients with unipolar and bipolar depression. Cox proportional hazards regression models examined the association between time-varying pramipexole exposure and substance use disorder risk using a counting process approach with multiple records per individual to accommodate time-varying exposure status. Robust variance estimation with cluster adjustments accounted for within-individual correlation across multiple observation periods.

Multivariable models included covariates that demonstrated statistical significance in univariable analyses. The proportional hazards assumption was evaluated using Schoenfeld residuals, and sensitivity analyses were performed with the variables that violated this assumption (p<0.05) entered as separate strata. Separate analyses were conducted for the overall cohort and stratified by mood disorder type (unipolar depression vs. bipolar disorder).

To assess the robustness of our findings, we conducted sensitivity analyses excluding patients with only a single pramipexole prescription, as these patients may not have initiated sustained treatment. These analyses were performed for all adjusted models (overall cohort and both diagnostic subgroups) to evaluate whether the observed associations persisted among patients with evidence of continued pramipexole use.

To assess the robustness of the association observed in the bipolar disorder subgroup to potential unmeasured confounding, we calculated E-values for the adjusted hazard ratio and the confidence interval limit closest to the null (22). The E-value indicates the minimum strength of association an unmeasured confounder would need with both exposure and outcome to fully explain the observed association.

In order to estimate the proportion of patients who were treated with pramipexole as psychiatric augmentation, we created a proxy measure consisting of psychiatric drugs (antidepressants or neuroleptics including lithium, ATC codes N06A and N05, respectively) being dispensed on the same date as the first pramipexole prescription.

All statistical analyses were conducted using R software version 4.5.2 (23), with statistical significance defined as p<0.05.

## Results

The study cohort comprised 3, 105 patients with unipolar or bipolar depression who received pramipexole treatment during the study period (**Table 1**). Baseline characteristics showed a predominance of female patients (68.4%, n=2, 123) across all age groups. The largest age group was 45–64 years (38.4%, n=1, 192), followed by ≥65 years (31.5%, n=978), 25–44 years (26.1%, n=811), and <25 years (4.0%, n=124). The majority of patients (78.2%, n=2, 427) had unipolar depression, while 21.8% (n=678) had bipolar disorder as their primary mood disorder diagnosis. Among individuals classified as having bipolar disorder, 50.4 percent (n = 342) received lithium, 84.5 percent (n = 573) received antidepressant treatment without lithium, and 35.0 percent (n = 237) received both lithium and an antidepressant at the index date or within 365 days before pramipexole initiation.

**Table 1:** Baseline characteristics of patients with mood disorders receiving pramipexole treatment.

Variables	n (%)	Unipolar, n (%)	Bipolar, n (%)	p value
Total	3105 (100)	2427 (78.2)	678 (21.8)	
Gender				0.401
Men	982 (31.6)	777 (32.0)	205 (30.2)	
Women	2123 (68.4)	1650 (68.0)	473 (69.8)	
Age categories				0.052
< 25	124 (4.0)	99 (4.1)	25 (3.7)	
25-44	811 (26.1)	612 (25.2)	199 (29.4)	
45-64	1192 (38.4)	926 (38.2)	266 (39.2)	
65+	978 (31.5)	790 (32.6)	188 (27.7)	
Comorbid anxiety disorders				0.461
No	1550 (49.9)	1203 (49.6)	347 (51.2)	
Yes	1555 (50.1)	1224 (50.4)	331 (48.8)	
Incident SUD event at follow-up				0.042
No	2715 (87.4)	2138 (88.1)	577 (85.1)	
Yes	390 (12.6)	289 (11.9)	101 (14.9)	
Incident SUD diagnosis at follow-up				0.008
No	2764 (89.0)	2180 (89.8)	584 (86.1)	
Yes	341 (11.0)	247 (10.2)	94 (13.9)	
Incident AUD medication at follow-up				1
No	2997 (96.5)	2342 (96.5)	655 (96.6)	
Yes	108 (3.5)	85 (3.5)	23 (3.4)	
Incident OUD medication at follow-up				0.197
No	3083 (99.3)	2407 (99.2)	676 (99.7)	
Yes	22 (0.7)	20 (0.8)	2 (0.3)	

Characteristics stratified by primary mood disorder diagnosis (unipolar vs. bipolar depression). SUD, substance use disorder; AUD, alcohol use disorder; OUD, opioid use disorder. Values presented as n (%) unless otherwise specified. P-values calculated using chi-square test or Fisher's exact test as appropriate.

Comorbid anxiety disorders were present in 50.1% (n=1, 555) of the cohort. During follow-up, 20, 683 person-years of observation were accumulated over a median follow-up period of 6.2 years (IQR: 2.9-10.1). Overall mortality during the study period was 21.4% (n=664).

A total of 390 patients (12.6%) developed incident substance use disorders during the observation period, corresponding to an incidence rate of 18.9 per 1, 000 person-years. Among patients with unipolar depression, 289 of 2, 427 (11.9%) developed substance use disorders, while 101 of 678 (14.9%) patients with bipolar disorder experienced this outcome.

Exposure analysis revealed that the majority of patients (2, 505 patients, 80.7%) were classified in the low exposure group during their complete follow-up period, representing minimal or intermittent pramipexole use. The intermediate exposure group comprised 271 patients (8.7%), while the high exposure group included 329 patients (10.6%). The distribution of observation time reflected this pattern, with 17, 583 person-years (85.1%) accumulated in the low exposure category, 1, 577 person-years (7.6%) in the intermediate exposure category, and 1, 524 person-years (7.4%) in the high exposure category. Among patients developing substance use disorders, 339 (86.9%) were in the low exposure group, 22 (5.6%) in the intermediate group, and 29 (7.4%) in the high exposure group.

Examining exposure progression over time among patients who remained at risk showed that most patients remained in the low exposure category throughout follow-up. Among the 2, 921 patients still at risk at 1 year, 2, 893 patients (99.0%) were classified as low exposure, with only 22 patients (0.8%) reaching intermediate exposure and 6 patients (0.2%) reaching high exposure levels. By 3 years, among the 2, 472 patients remaining at risk, the distribution had shifted somewhat, with 2, 195 patients (88.8%) remaining in the low exposure group, while 223 patients (9.0%) had progressed to intermediate exposure and 54 patients (2.2%) to high exposure. After 5 years of follow-up, among the 2, 037 patients still at risk, 1, 663 patients (81.6%) remained in the low exposure category, 226 patients (11.1%) were classified as intermediate exposure, and 148 patients (7.3%) had reached high cumulative exposure levels.

Multivariable Cox regression analysis of the entire cohort identified several significant risk factors for substance use disorder development (**Table 2**). Female gender was associated with reduced risk compared to males, though this did not reach statistical significance (adjusted HR 0.92, 95% CI: 0.74-1.14, p=0.441). Increasing age showed a protective effect (adjusted HR 0.98 per year, 95% CI: 0.98-0.99, p<0.001).

**Table 2:** Cox proportional hazards regression analysis of substance use disorder risk by pramipexole exposure categories.

Variables	n (%)	Events (n)	uHR (95% CI)	aHR (95% CI)	p value
Gender					
Men	982	123	1	1	–
Women	2123	267	0.95 (0.77-1.18)	0.92 (0.74-1.14)	0.441
Age (per year)	3105	390	0.98 (0.98-0.99)	0.98 (0.98-0.99)	< 0.001
Bipolar disorder					
No	2427	289	1	1	–
Yes	678	101	1.37 (1.10-1.72)	1.36 (1.09-1.71)	0.007
Anxiety disorders					
No	1550	171	1	1	–
Yes	1555	219	1.48 (1.21-1.81)	1.33 (1.08-1.63)	0.007
Pramipexole dose category					
Low	2505	339	1	1	–
Intermediate	271	22	0.90 (0.58-1.40)	0.93 (0.60-1.45)	0.759
High	329	29	1.43 (0.96-2.12)	1.51 (1.02-2.23)	0.042

Results from Cox regression models examining association between pramipexole dose categories and incident substance use disorder. uHR, unadjusted hazard ratio; aHR, adjusted hazard ratio. Dose categories: Low = 0 to <1 daily dose equivalent per year, Intermediate = 1 to <2 daily dose equivalents per year, High = 2+ daily dose equivalents per year. For time-varying pramipexole dose categories, n represents the number of patients who ever experienced each exposure level during follow-up. P-values from adjusted models.

Psychiatric comorbidities demonstrated strong associations with substance use disorder risk. Bipolar disorder was associated with a 36% increased risk compared to unipolar depression (adjusted HR 1.36, 95% CI: 1.09-1.71, p=0.007). Comorbid anxiety disorders conferred a 33% increased risk (adjusted HR 1.33, 95% CI: 1.08-1.63, p=0.007).

Pramipexole exposure demonstrated a significant dose-response relationship with substance use disorder risk in the overall cohort. Compared to the lowest exposure group, intermediate exposure showed no significant association (adjusted HR 0.93, 95% CI: 0.60-1.45, p=0.759), while high exposure demonstrated a significantly increased risk (adjusted HR 1.51, 95% CI: 1.02-2.23, p=0.042) (**Table 2**).

Among patients with unipolar depression (n=2, 427), no significant dose-response relationship was observed. Intermediate exposure showed adjusted HR 1.03 (95% CI: 0.63-1.67, p=0.916), while high exposure demonstrated a non-significant association (adjusted HR 1.23, 95% CI: 0.76-2.00, p=0.396). Risk factors in the unipolar depression subgroup included increasing age (protective effect: adjusted HR 0.98, 95% CI: 0.98-0.99, p<0.001) and comorbid anxiety disorders (increased risk: adjusted HR 1.29, 95% CI: 1.01-1.64, p=0.042) (**Table 3**). Gender violated the proportional hazards assumption in this subgroup (p=0.044); a stratified analysis addressing this violation yielded similar results for the other covariates (**Supplementary Table 1**). In the bipolar disorder subgroup (n=678), a markedly different pattern emerged. While intermediate exposure showed no significant association (adjusted HR 0.68, 95% CI: 0.25-1.86, p=0.452), high exposure demonstrated a significantly increased risk of substance use disorders (adjusted HR 2.58, 95% CI: 1.27-5.24, p=0.009) compared to low exposure. Risk factors in the bipolar disorder subgroup included increasing age (protective effect: adjusted HR 0.98, 95% CI: 0.97-1.00, p=0.013) (**Table 4**). Comorbid anxiety disorders violated the proportional hazards assumption in this subgroup; a stratified analysis addressing this violation yielded virtually identical results for pramipexole dose categories, with high exposure maintaining a significantly increased risk (adjusted HR 2.54, 95% CI: 1.25-5.16, p=0.010) (**Supplementary Table 2**). In the bipolar disorder subgroup, the high-exposure category included 65 individuals, among whom 11 incident substance use disorder events were identified. The association for high exposure was statistically significant but imprecisely estimated, as reflected by the wide confidence interval (adjusted HR 2.54, 95% CI 1.25–5.16)”.

**Table 3:** Cox proportional hazards regression analysis of substance use disorder risk in patients with unipolar depression.

Variables	n (%)	Events (n)	uHR (95% CI)	aHR (95% CI)	p value
Gender					
Men	777	99	1	1	–
Women	1650	190	0.85 (0.67-1.09)	0.85 (0.66-1.08)	0.184
Age (per year)	2427	289	0.98 (0.97-0.99)	0.98 (0.98-0.99)	< 0.001
Anxiety disorders					
No	1203	126	1	1	–
Yes	1224	163	1.45 (1.15-1.82)	1.28 (1.00-1.63)	0.046
Pramipexole dose category					
Low	1944	253	1	1	–
Intermediate	219	18	1.00 (0.61-1.62)	1.03 (0.63-1.68)	0.904
High	264	18	1.15 (0.71-1.86)	1.20 (0.74-1.95)	0.453

Subgroup analysis restricted to patients with unipolar depression (n=2,427). uHR = unadjusted hazard ratio; aHR, adjusted hazard ratio. Dose categories: Low = 0 to <1 daily dose equivalent per year, Intermediate = 1 to <2 daily dose equivalents per year, High = 2+ daily dose equivalents per year. For time-varying pramipexole dose categories, n represents the number of patients who ever experienced each exposure level during follow-up. P-values from adjusted models.

**Table 4:** Cox proportional hazards regression analysis of substance use disorder risk in patients with bipolar disorder.

Variables	n (%)	Events (n)	uHR (95% CI)	aHR (95% CI)	p value
Gender					
Men	205	24	1	1	–
Women	473	77	1.34 (0.85-2.11)	1.20 (0.75-1.92)	0.449
Age (per year)	678	101	0.98 (0.97-0.99)	0.98 (0.97-1.00)	0.012
Anxiety disorders					
No	347	45	1	1	–
Yes	331	56	1.65 (1.12-2.43)	1.44 (0.96-2.14)	0.075
Pramipexole dose category					
Low	561	86	1	1	–
Intermediate	52	4	0.64 (0.23-1.75)	0.70 (0.25-1.92)	0.490
High	65	11	2.37 (1.17-4.78)	2.54 (1.25-5.16)	0.010

Subgroup analysis restricted to patients with bipolar disorder (n=678). uHR = unadjusted hazard ratio; aHR, adjusted hazard ratio. Dose categories: Low = 0 to <1 daily dose equivalent per year, Intermediate = 1 to <2 daily dose equivalents per year, High = 2+ daily dose equivalents per year. For time-varying pramipexole dose categories, n represents the number of patients who ever experienced each exposure level during follow-up. P-values from adjusted models.

To evaluate the robustness of our findings, we conducted sensitivity analyses excluding patients with only a single pramipexole prescription (n=1, 050 excluded), resulting in 2, 055 patients with evidence of sustained treatment (**Supplementary Tables 3**–**S5**). The pattern of results remained largely consistent with the main analysis. Among patients with unipolar depression (n=1, 595), no significant dose-response relationship was observed, with high exposure showing adjusted HR 1.20 (95% CI: 0.72-1.99, p=0.484). Most importantly, the key finding in bipolar disorder patients was preserved: High pramipexole exposure remained significantly associated with increased substance use disorder risk (adjusted HR 2.28, 95% CI: 1.07-4.85, p=0.032). These sensitivity analyses support the robustness of our main findings, particularly the differential dose-response relationship between unipolar depression and bipolar disorder patients.

For the association between high cumulative pramipexole exposure and incident substance use disorder in the bipolar disorder subgroup, the E-value was 4.52 for the point estimate and 1.81 for the lower confidence limit.

The proportion of patients who were prescribed other psychiatric drugs at the same date as the first pramipexole prescription was 25.9 percent of patients with depressive disorders (n = 629) and 26.7 percent of patients with bipolar disorders (n = 181). The proportion of bipolar patients in the highest pramipexole dose group was 23.1 percent (n = 15).

## Discussion

In this nationwide cohort of individuals treated for mood disorders within specialised mental health care, high cumulative exposure to pramipexole was associated with an increased risk of incident substance use disorder. The association was particularly pronounced among individuals with bipolar disorder. In contrast, no clear exposure-risk gradient was observed among patients with unipolar depression. These findings raise the possibility that prolonged pramipexole exposure may be associated with increased risk of substance use disorder in some patient groups, particularly among individuals with bipolar disorder. However, alternative explanations, including differences in underlying illness characteristics and treatment selection, cannot be excluded. In the overall cohort, the observed association was modest in magnitude and primarily evident at higher levels of cumulative exposure, suggesting a potential threshold effect rather than a linear exposure-risk relationship.

Existing evidence regarding pramipexole in mood disorders has largely been derived from clinical trials focusing on short-term efficacy and tolerability. While such trials are essential for establishing antidepressant effects and short-term safety, they may have limited ability to detect uncommon outcomes or outcomes that develop over a longer time span, requiring prolonged follow-up. In this context, the present nationwide register-based study complements trial evidence by capturing long-term, cumulative exposure in routine psychiatric care.

Substance use disorders share key neurobiological and behavioural features with impulse control disorders associated with dopamine agonists, including compulsivity, diminished top-down control, and heightened responsiveness to reward cues, despite differences in primary reinforcers (14–16, 24). More broadly, dopaminergic agents have also been investigated as therapeutic options in patients with severe mental illness and established substance use disorders (25). However, such studies address a different clinical question and do not exclude the possibility that sustained dopamine agonist exposure may affect addiction vulnerability in other psychiatric populations. While impulse control disorders such as gambling disorder have been most extensively studied in Parkinson’s disease populations treated with dopamine agonists, where substance use disorders have not emerged as a prominent adverse outcome (26, 27), the present findings raise the possibility that dopaminergic behavioural risks may also be relevant to substance-related outcomes in psychiatric populations. Importantly, bipolar disorder was itself independently associated with elevated substance use disorder risk in this cohort, underscoring its role as a vulnerability context in which additional pharmacological risk factors may have amplified effects. The finding that the association between high pramipexole exposure and incident substance use disorder was most pronounced in individuals with bipolar disorder is clinically and theoretically consistent with existing literature. Bipolar disorder is characterised by earlier initiation of substance use, elevated baseline rates of substance use disorders, and more severe addiction-related outcomes compared with unipolar depression (2, 28).

The stronger association observed in individuals with bipolar disorder should be interpreted against the background of their already elevated vulnerability to substance-related outcomes. Bipolar disorder is characterised by a high burden of psychiatric comorbidity, including substance use disorders, anxiety disorders and ADHD, and comorbid substance use disorder has been associated with a more complex illness course, poorer treatment adherence and increased risk of suicide attempts (29). Clinical characteristics associated with substance-related comorbidity in bipolar disorder, including earlier illness onset, psychotic or residual symptoms and psychiatric family history, were not available in the present study and may have contributed to residual confounding. Thus, the stronger association in the bipolar subgroup may partly reflect underlying clinical vulnerability and treatment selection rather than a differential pharmacological effect of pramipexole alone.

Dopaminergic sensitivity is thought to be heightened in bipolar disorder, particularly during manic and hypomanic states, but also potentially as a more enduring trait-like feature, independent of acute mood state, in line with evidence of increased reward responsivity and motivational drive in bipolar disorder (30). In this context, pramipexole-induced dopaminergic stimulation may further potentiate reward-driven and disinhibited behaviours, increasing susceptibility to substance-related addictive disorders. Reports of treatment-emergent hypomanic or manic symptoms in pramipexole trials for bipolar depression support the notion that dopaminergic augmentation can precipitate behavioural activation in this population, which may in turn facilitate escalation of substance use (6, 12).Notably, increased substance use disorder risk was selectively observed among individuals with high cumulative pramipexole exposure, whereas intermediate exposure levels were not associated with elevated risk, suggesting that sustained dopaminergic stimulation may be required before clinically detectable substance-related outcomes emerge. Such patterns are consistent with sensitisation models of addiction, in which repeated dopaminergic stimulation leads to enduring changes in reward circuitry and behavioural responsiveness (31).

Co-prescription timing provides a tentative proxy, indicating that at least about 25 percent of patients in the present study might be prescribed pramipexole as psychiatric augmentation. However, the accuracy of this proxy measure is questionable. First, it cannot be taken for granted that prescriptions of other drugs will take place at the same time as the first pramipexole prescription. Second, the present study is based on dispensation dates rather than prescription dates, and it cannot be assumed that most patients purchase other drugs at the specific date when they purchase their first pramipexole prescription. Third, data on lamotrigine and valproic acid were not available in the data set used in the present study, further increasing the risk of under-ascertainment. Further studies should examine prescriber specialisation, which is data that is available in the PDR, albeit not available in the data set used in the present study.

An important question is why substance use disorder has not emerged as a prominent adverse outcome in previous studies of pramipexole in mood disorders. Several explanations are possible. First, most clinical trials have focused on antidepressant efficacy and short- to medium-term safety, with follow-up periods typically ranging from weeks to months rather than years. Second, trial participants are often carefully selected and monitored, potentially reducing the likelihood of behavioural complications being missed or progressing to clinically significant substance use disorders. In addition, participants in clinical trials typically undergo regular clinical assessments, including monitoring for behavioural adverse effects and emerging psychiatric symptoms. Such monitoring may facilitate early detection and management of problematic behaviours, potentially preventing progression to clinically significant substance use disorders. In contrast, the present study reflects routine clinical practice, where the intensity and frequency of monitoring are likely to vary considerably. Third, the present study examined cumulative exposure over prolonged periods in routine clinical practice, thereby capturing patterns of treatment and patient populations that may differ substantially from those represented in clinical trials. Consequently, differences between the present findings and previous studies do not necessarily represent contradictory evidence, but may reflect the ability of different study designs to detect different types of outcomes.

The present findings should be regarded as hypothesis-generating and require replication in studies with more detailed clinical information and designs better suited for causal inference. Future clinical trials of pramipexole in mood disorders should consider systematic assessment of substance use outcomes alongside other behavioural adverse effects.

### Strengths and limitations

The strengths of this study include its nationwide register-based design, large sample size, long follow-up, and longitudinal assessment of cumulative pramipexole exposure. Modelling exposure as a time-varying variable reduced the risk of immortal time bias, while the use of incident substance use disorder outcomes allowed the temporal relationship between exposure and outcome to be examined. Sensitivity analyses excluding individuals with only a single pramipexole prescription yielded similar estimates, with the association for high cumulative exposure in the bipolar disorder subgroup remaining statistically significant.

Several limitations should be considered. First, as in other observational pharmacoepidemiological studies, the findings may be affected by confounding by indication and treatment selection. Individuals reaching high cumulative pramipexole exposure may have differed systematically from those with limited exposure. For example, they may have had more severe, recurrent or treatment-resistant illness, incomplete treatment response, greater behavioural dysregulation, or a higher underlying susceptibility to addictive disorders. The registers did not contain information on current mood state, mixed or hypomanic symptoms, treatment response, impulsivity, craving, personality traits, family history of substance use disorder, subthreshold substance use, ADHD symptoms not reflected in registered diagnoses, or other relevant clinical and behavioural characteristics. These factors could be associated with both prolonged pramipexole treatment and subsequent substance use disorder and may therefore have contributed to residual confounding.

The E-value analysis indicated that substantial unmeasured confounding would be required to fully explain the point estimate for high cumulative exposure in the bipolar disorder subgroup. However, the lower confidence limit was less robust to unmeasured confounding, reinforcing the need for cautious interpretation and replication in studies with more detailed clinical information.

Furthermore, the clinical indication for pramipexole could not be determined. Although inclusion required a registered diagnosis of unipolar depression or bipolar disorder and treatment with an antidepressant or lithium at the index date or within 365 days prior to that date, these criteria do not establish that pramipexole was prescribed as psychiatric augmentation. Pramipexole may instead have been prescribed for a somatic condition such as restless legs syndrome or fibromyalgia. To obtain an approximate indication of whether pramipexole was initiated in the context of psychiatric pharmacotherapy, we constructed an exploratory proxy based on same-day dispensing of pramipexole and other psychiatric medications. Same-day co-dispensation was identified in approximately one-quarter of both diagnostic groups and in 23.1% of individuals with bipolar disorder who reached the high cumulative exposure category. This indicates that pramipexole was dispensed in direct temporal proximity to other psychiatric pharmacotherapy in a subset of the cohort but does not establish the treatment indication.

The proxy measure has important limitations. Its sensitivity is likely to be limited because initiation of pramipexole does not necessarily coincide with the renewal or collection of existing psychiatric medications. Its specificity for psychiatric augmentation is also uncertain because the Prescribed Drug Register records pharmacy dispensing rather than prescribing dates. Medications prescribed concurrently may therefore be collected on different dates, while medications dispensed on the same date are not necessarily prescribed for the same condition. Consequently, the proxy should be interpreted as a tentative indicator of psychiatric co-treatment rather than a reliable measure of the indication for pramipexole. Information on prescriber speciality, which is available in the Prescribed Drug Register but was not included in the dataset used for the present study, could potentially provide a more informative proxy in future research.

Second, the bipolar disorder subgroup may not be fully representative of the broader population with bipolar disorder. Inclusion required treatment with an antidepressant or lithium, whereas data on lamotrigine and valproate were not available in the present dataset. Antipsychotic medications, including quetiapine, were not used as inclusion criteria because their multiple possible indications prevented reliable identification of treatment for bipolar depression. Patients treated exclusively with lamotrigine, valproate or antipsychotic-based mood-stabilising regimens may therefore have been omitted. This may limit the generalisability of the findings to the broader population of individuals with bipolar disorder. The absence of lamotrigine and valproate data may also have reduced the sensitivity of the same-day co-dispensation proxy for identifying psychiatric co-treatment.

Third, outcome ascertainment was restricted to substance use disorder diagnoses recorded in specialised healthcare and dispensed medications used in the treatment of alcohol or opioid use disorder. Substance use disorders that were present but not recorded at baseline may therefore have been misclassified as incident outcomes during follow-up. The direction of any resulting bias is uncertain and would depend on whether unrecognised baseline substance use disorder was associated with subsequent cumulative pramipexole exposure. Conversely, incident substance-related problems that did not result in specialised healthcare or pharmacological treatment were not identified. The outcomes captured in this study are therefore likely to represent the more clinically recognised or severe end of the spectrum of substance-related problems.

Differential detection may also have influenced the findings. Individuals receiving prolonged pramipexole treatment and consequently reaching higher cumulative exposure may have had more frequent or sustained contact with psychiatric services. This could have provided more opportunities for an existing or emerging substance-related problem to be recognised and formally recorded. If the intensity of clinical contact differed according to cumulative exposure, differential detection could have contributed to the observed association independently of any pharmacological effect.

Fourth, cumulative pramipexole exposure was estimated from dispensed prescriptions and does not establish that the medication was taken as prescribed. Moreover, the cumulative-exposure measure could not distinguish between a higher daily dose administered over a shorter period and a lower daily dose administered over a longer period. Although cumulative exposure was selected to capture differences in the total amount of pramipexole dispensed, it does not provide a direct measure of either treatment duration or actual daily dose. The observed pattern should therefore not be interpreted as demonstrating a biological dose-response relationship.

Finally, the association in the bipolar high-exposure category was based on a relatively small number of individuals and events, as reflected by the wide confidence interval. The magnitude and diagnostic specificity of this association should therefore be interpreted cautiously.

Taken together, the observational design, potential residual confounding, uncertain treatment indication, limitations in cohort selection, possible outcome misclassification and limited precision of the subgroup estimate preclude causal interpretation. The findings should be considered hypothesis-generating and require replication in studies with more detailed information on treatment indication, prescriber speciality, daily dose, treatment duration, treatment response, healthcare contact and established risk factors for substance use disorder. Where sufficiently detailed longitudinal data are available, future studies could use research designs explicitly aimed at strengthening causal inference, such as target trial emulation, to address treatment selection, time-varying exposure and relevant confounding more systematically (32). Prospective studies and clinical trials that repeatedly assess substance use, craving, impulsivity and reward-seeking behaviour during pramipexole treatment would be particularly valuable.

## Clinical implications and conclusions

From a clinical perspective, these findings highlight the importance of careful risk-benefit assessment when prescribing pramipexole to individuals with mood disorders, particularly those with bipolar disorders or other indicators of addiction vulnerability. In addition to the diagnostic subgroup, younger age and comorbid anxiety disorders emerged as relevant modifiers of substance use disorder risk, suggesting that vulnerability extends beyond diagnosis alone. While pramipexole may offer antidepressant benefits as an augmentation strategy, clinicians should be aware of potential behavioural adverse effects beyond impulse control disorders, including the possible emergence of substance use disorders. Notably, substance use disorder has not emerged as a prominent or systematically reported adverse outcome in Parkinson’s disease populations treated with dopamine agonists, despite extensive clinical experience and documentation of other behavioural complications such as impulse control disorders (26, 27). The present findings therefore suggest that dopaminergic exposure may confer qualitatively different behavioural risks in psychiatric populations, particularly in individuals with major depressive disorder or bipolar disorder. Systematic monitoring for changes in substance use, craving, and reward-seeking behaviour may therefore be warranted during dopaminergic treatment, particularly during dose escalation or prolonged exposure at higher cumulative doses. Such monitoring could include proactive screening for substance-related problems, patient and family education regarding behavioural risks, and consideration of alternative treatment strategies in individuals with elevated baseline risk In future research, prospective studies and clinical trials should systematically assess changes in substance use, craving, impulsivity, and reward-seeking behaviour during pramipexole treatment.

In conclusion, cumulative exposure to pramipexole was associated with an increased risk of incident substance use disorder in this nationwide cohort of individuals with mood disorders, with the strongest associations observed among patients with bipolar disorder and evidence of a dose-response pattern primarily at higher exposure levels. Although these findings require confirmation in further studies, they underscore the need to consider behavioural and addictive risks when prescribing dopaminergic agents in psychiatric populations and support enhanced monitoring strategies in clinically vulnerable groups.

## Data Availability

The raw data supporting the conclusions of this article are not publicly available due to data protection regulations. Data access requests can be directed to the National Board of Health and Welfare.se.
