# Supplementary material for: Pramipexole treatment and substance use disorder risk in unipolar and bipolar depression: a Swedish nationwide register study
Source: Front Psychiatry. 2026 Aug 10;17:1919633. doi: 10.3389/fpsyt.2026.1919633 (PMC13500636; doi:10.3389/fpsyt.2026.1919633)
Supplement: Supplementary file 1 [file SupplementaryFile1.docx]

# Supplementary Tables

| **Table S1** | |  |  |  |  |  |
| --- | --- | --- | --- | --- | --- | --- |
| *Cox Proportional Hazards Regression Analysis of Substance Use Disorder Risk in Patients with Unipolar Depression - Stratified by Sex* | | | | | | |
| Variables | | n (%) | Events (n) | uHR (95% CI) | aHR (95% CI) | p value |
| Age (per year) | | 2427 | 289 | 0.98 (0.97-0.99) | 0.98 (0.98-0.99) | < 0.001 |
| Anxiety disorders | |  |  |  |  |  |
|  | No | 1203 | 126 | 1 | 1 | - |
|  | Yes | 1224 | 163 | 1.46 (1.16-1.84) | 1.29 (1.01-1.64) | 0.042 |
| Pramipexole dose category | |  |  |  |  |  |
|  | Low | 1944 | 253 | 1 | 1 | - |
|  | Intermediate | 219 | 18 | 0.99 (0.61-1.61) | 1.03 (0.63-1.67) | 0.916 |
|  | High | 264 | 18 | 1.16 (0.72-1.89) | 1.23 (0.76-2.00) | 0.396 |
| *Stratified analysis addressing proportional hazards assumption violation for sex (p=0.044). Results stratified by sex with shared baseline hazard function. uHR = unadjusted hazard ratio; aHR = adjusted hazard ratio. Dose categories: Low = 0 to <1 daily dose equivalent per year, Intermediate = 1 to <2 daily dose equivalents per year, High = 2+ daily dose equivalents per year. P-values from adjusted models.* | | | | | | |

| **Table S2** | |  |  |  |  |  |
| --- | --- | --- | --- | --- | --- | --- |
| *Cox Proportional Hazards Regression Analysis of Substance Use Disorder Risk in Patients with Bipolar Disorder - Stratified by Anxiety Disorders* | | | | | | |
| Variables | | n (%) | Events (n) | uHR (95% CI) | aHR (95% CI) | p value |
| Gender | |  |  |  |  |  |
|  | Men | 205 | 24 | 1 | 1 | - |
|  | Women | 473 | 77 | 1.25 (0.79-1.97) | 1.19 (0.74-1.90) | 0.479 |
| Age (per year) | | 678 | 101 | 0.98 (0.97-1.00) | 0.98 (0.97-1.00) | 0.013 |
| Pramipexole dose category | |  |  |  |  |  |
|  | Low | 561 | 86 | 1 | 1 | - |
|  | Intermediate | 52 | 4 | 0.64 (0.23-1.76) | 0.68 (0.25-1.86) | 0.452 |
|  | High | 65 | 11 | 2.41 (1.18-4.91) | 2.58 (1.27-5.24) | 0.009 |
| *Stratified analysis addressing proportional hazards assumption violation for anxiety disorders. Results stratified by anxiety disorder status with shared baseline hazard function. uHR = unadjusted hazard ratio; aHR = adjusted hazard ratio. Dose categories: Low = 0 to <1 daily dose equivalent per year, Intermediate = 1 to <2 daily dose equivalents per year, High = 2+ daily dose equivalents per year. P-values from adjusted models.* | | | | | | |

| **Table S3** | |  |  |  |  |  |
| --- | --- | --- | --- | --- | --- | --- |
| *Cox Proportional Hazards Regression Analysis of Substance Use Disorder Risk by Pramipexole Exposure Categories - Sensitivity Analysis Excluding Single-Prescription Patients* | | | | | | |
| Variables | | n (%) | Events (n) | uHR (95% CI) | aHR (95% CI) | p value |
| Gender | |  |  |  |  |  |
|  | Men | 648 | 83 | 1 | 1 | - |
|  | Women | 1407 | 186 | 1.01 (0.78-1.31) | 0.97 (0.74-1.25) | 0.794 |
| Age (per year) | | 2055 | 269 | 0.98 (0.97-0.99) | 0.98 (0.97-0.99) | < 0.001 |
| Bipolar disorder | |  |  |  |  |  |
|  | No | 1595 | 199 | 1 | 1 | - |
|  | Yes | 460 | 70 | 1.30 (0.99-1.71) | 1.27 (0.97-1.67) | 0.084 |
| Anxiety disorders | |  |  |  |  |  |
|  | No | 1029 | 116 | 1 | 1 | - |
|  | Yes | 1026 | 153 | 1.54 (1.21-1.96) | 1.38 (1.08-1.77) | 0.011 |
| Pramipexole dose category | |  |  |  |  |  |
|  | Low | 1455 | 218 | 1 | 1 | - |
|  | Intermediate | 271 | 22 | 0.88 (0.56-1.38) | 0.91 (0.58-1.43) | 0.694 |
|  | High | 329 | 29 | 1.35 (0.89-2.05) | 1.45 (0.95-2.20) | 0.082 |
| *Sensitivity analysis restricted to patients with multiple pramipexole prescriptions (n=2,055). uHR = unadjusted hazard ratio; aHR = adjusted hazard ratio. Pramipexole exposure categories: Low = 0 to <1 daily dose equivalent per year, Intermediate = 1 to <2 daily dose equivalents per year, High = 2+ daily dose equivalents per year. P-values from adjusted models.* | | | | | | |

| **Table S4** | |  |  |  |  |  |
| --- | --- | --- | --- | --- | --- | --- |
| *Cox Proportional Hazards Regression Analysis of Substance Use Disorder Risk in Patients with Unipolar Depression - Sensitivity Analysis Excluding Single-Prescription Patients* | | | | | | |
| Variables | | n (%) | Events (n) | uHR (95% CI) | aHR (95% CI) | p value |
| Gender | |  |  |  |  |  |
|  | Men | 513 | 68 | 1 | 1 | - |
|  | Women | 1082 | 131 | 0.88 (0.66-1.19) | 0.88 (0.65-1.18) | 0.386 |
| Age (per year) | | 1595 | 199 | 0.98 (0.97-0.99) | 0.98 (0.97-0.99) | < 0.001 |
| Anxiety disorders | |  |  |  |  |  |
|  | No | 791 | 84 | 1 | 1 | - |
|  | Yes | 804 | 115 | 1.53 (1.16-2.02) | 1.36 (1.02-1.82) | 0.035 |
| Pramipexole dose category | |  |  |  |  |  |
|  | Low | 1112 | 163 | 1 | 1 | - |
|  | Intermediate | 219 | 18 | 1.00 (0.61-1.66) | 1.03 (0.62-1.71) | 0.900 |
|  | High | 264 | 18 | 1.13 (0.68-1.87) | 1.20 (0.72-1.99) | 0.484 |
| *Sensitivity analysis restricted to patients with unipolar depression and multiple pramipexole prescriptions (n=1,595). uHR = unadjusted hazard ratio; aHR = adjusted hazard ratio. Pramipexole exposure categories: Low = 0 to <1 daily dose equivalent per year, Intermediate = 1 to <2 daily dose equivalents per year, High = 2+ daily dose equivalents per year. P-values from adjusted models.* | | | | | | |

| **Table S5** | |  |  |  |  |  |
| --- | --- | --- | --- | --- | --- | --- |
| *Cox Proportional Hazards Regression Analysis of Substance Use Disorder Risk in Patients with Bipolar Disorder - Sensitivity Analysis Excluding Single-Prescription Patients* | | | | | | |
| Variables | | n (%) | Events (n) | uHR (95% CI) | aHR (95% CI) | p value |
| Gender | |  |  |  |  |  |
|  | Men | 135 | 15 | 1 | 1 | - |
|  | Women | 325 | 55 | 1.55 (0.89-2.72) | 1.35 (0.75-2.41) | 0.318 |
| Age (per year) | | 460 | 70 | 0.98 (0.97-0.99) | 0.98 (0.97-1.00) | 0.033 |
| Anxiety disorders | |  |  |  |  |  |
|  | No | 238 | 32 | 1 | 1 | - |
|  | Yes | 222 | 38 | 1.63 (1.03-2.6) | 1.36 (0.83-2.22) | 0.220 |
| Pramipexole dose category | |  |  |  |  |  |
|  | Low | 343 | 55 | 1 | 1 | - |
|  | Intermediate | 52 | 4 | 0.59 (0.21-1.65) | 0.68 (0.24-1.90) | 0.457 |
|  | High | 65 | 11 | 2.05 (0.97-4.34) | 2.28 (1.07-4.85) | 0.032 |
| *Sensitivity analysis restricted to patients with bipolar disorder and multiple pramipexole prescriptions (n=460). uHR = unadjusted hazard ratio; aHR = adjusted hazard ratio. Pramipexole exposure categories: Low = 0 to <1 daily dose equivalent per year, Intermediate = 1 to <2 daily dose equivalents per year, High = 2+ daily dose equivalents per year. P-values from adjusted models.* | | | | | | |
